# Pancreaticojejunostomy—a review of modern techniques

**DOI:** 10.1007/s00423-020-01855-6

**Published:** 2020-01-23

**Authors:** Marek Olakowski, Ewa Grudzińska, Sławomir Mrowiec

**Affiliations:** Department of Gastrointestinal Surgery, Medical University of Silesia, Katowice, Poland

**Keywords:** Pancreaticoduodenectomy, Pancreaticojejunostomy, Pancreatic fistula, Anastomosis

## Abstract

**Background:**

Pancreaticojejunal anastomosis is one of the most demanding procedures in surgery. Up to now, no technique has been proven to reduce the incidence of POPF when compared to the other methods.

**Purpose:**

The aim of this review was to provide a concise and illustrated description of the most recent methods of pancreaticojejunostomy. Their development was directly related to the still ongoing search by surgeons for such a technique of anastomosis that would eliminate the problem of POPF.

**Conclusions:**

Knowledge of various techniques of anastomosis may help the surgeon to find the most suitable and optimal method of pancreatic-intestinal anastomosis for the patient.

## Introduction

Pancreaticoduodenectomy (PD) is one of the greatest challenges in gastrointestinal surgery, with mortality < 5% in high-volume centers, and even 50% of perioperative complications [1, 2]. The anastomosis of the pancreatic stump is considered the most difficult phase of the surgery, crucial for postoperative healing. Technical failure at this step causes postoperative pancreatic fistula (POPF), a potentially fatal complication, with almost 26% mortality in its most severe type C cases [3].

There have been attempts to close the main pancreatic duct (MPD) by ligation, stapler or glue instead of performing an anastomosis. These methods have proven to be clinically ineffective, because closure of the main pancreatic duct (MPD) caused postoperative pancreatitis, equally lethal as a fistula [4].

Another way of avoiding POPF is a total pancreatectomy. The removal of the pancreatic stump is performed rarely, only when the resection margins are cancer positive or when the cancer is multifocal. This procedure is also acceptable in extremely high-risk patients, in whom according to an experienced surgeon, the risk of a POPF is very high (soft pancreatic tissue, MPD < 3 mm) [5–7]. In some centers, in order to prevent the difficult to manage postoperative diabetes, an auto-transplantation of pancreatic islets is additionally proposed [8].

Regardless of the POPF risk, an anastomosis between the pancreatic stump and the gastrointestinal tract remains the most effective and safe method of securing the remaining pancreas as it preserves the exocrine and endocrine function of the gland and guarantees the best quality of life after surgery.

Nowadays, two types of anastomoses are performed between the pancreas and the gastrointestinal tract:

1. pancreaticogastrostomy: between the pancreatic stump or the MPD and the stomach,
2. pancreaticojejunostomy: between the pancreatic stump or the MPD and the small intestine.

Pancreaticojejunal anastomosis was popularized by Whipple in the 1940s, when he resigned from the pancreatic stump ligation [9]. Even though it has been almost 80 years since the first pancreaticojejunal anastomosis, we are still lacking one, universally accepted technique. The POPF is called the “Achilles’ heel” of PD and pancreatic anastomoses require further improvement, as is reflected by extensive number of publications on this topic [10]. This paper presents a subjective review of the most interesting modern techniques of pancreaticojejunal anastomoses that have been published in recent years. The POPF rates of these techniques are presented in Table 1. As no technique has proven superior to others concerning the POPF rate [11], it seems that the anastomosis should be individually planned with regard both for the patient’s characteristics and for the surgeon’s preferences.

**Table 1:** POPF rates in different pancreaticojejunal anastomosis techniques

Reference number, author, year of publication	Number of patients	POPF rate (%)
Invagination techniques		
[10] Yang et al., 2017	22	0
[13] Peng et al., 2003	227	0
[14] Casadei et al., 2013	69	18.8
[15] Buc et al., 2010	45	8.9
[16] Kim et al., 2014	21	23.8
[17] Kim et al., 2016	42	0
[18] Li et al., 2015	23	8.7
[19] Kelemen et al., 2016	49	4.1
[20] Li et al., 2018	188	5.3
[21] Hashimoto et al., 2013	4	0
[22] Kuśnierz et al., 2015	52	1.9
[23] Gupta et al., 2018	32	0
[24] Chen et al., 2014	264	4.2
[25] Cho et al., 2014	15	20
[26] Kwon et al., 2015	134	38.8
[27] Yang et al., 2018	33	12.1
[28] Yao et al., 2016	16	12.5
[29] Katoh et al., 2013	34	15
[30] Liu et al., 2018	81	6.1
[31] Morelli et al., 2017	100	7
Duct-to-mucosa techniques		
[35] Torres et al., 2017	17	23.5
[36] Palampros et al., 2016	248	4.2
[37] Su et al., 2014	96	4.2
[38] Zhang et al., 2015	22	4.5
[41] Kim et al., 2017	151	37.1
[42] Chen et al., 2014	120	7.5
[43] Ji et al., 2015	35	17.1
[44] Grobmyer et al., 2010	187	6.9
[51] Kojima et al., 2018	101	2.9
[52] Wang et al., 2017	52	3.8

## Invaginating techniques

### Standard surgical technique

Anastomosis of the pancreatic stump is classically performed by invagination of 1–2 cm of the proximal end of the stump into the jejunum, end-to-end or end-to-side. This technique is recommended for patients with a narrow pancreatic duct (< 3 mm) and soft pancreatic tissue [12].

**Fig. 1:**
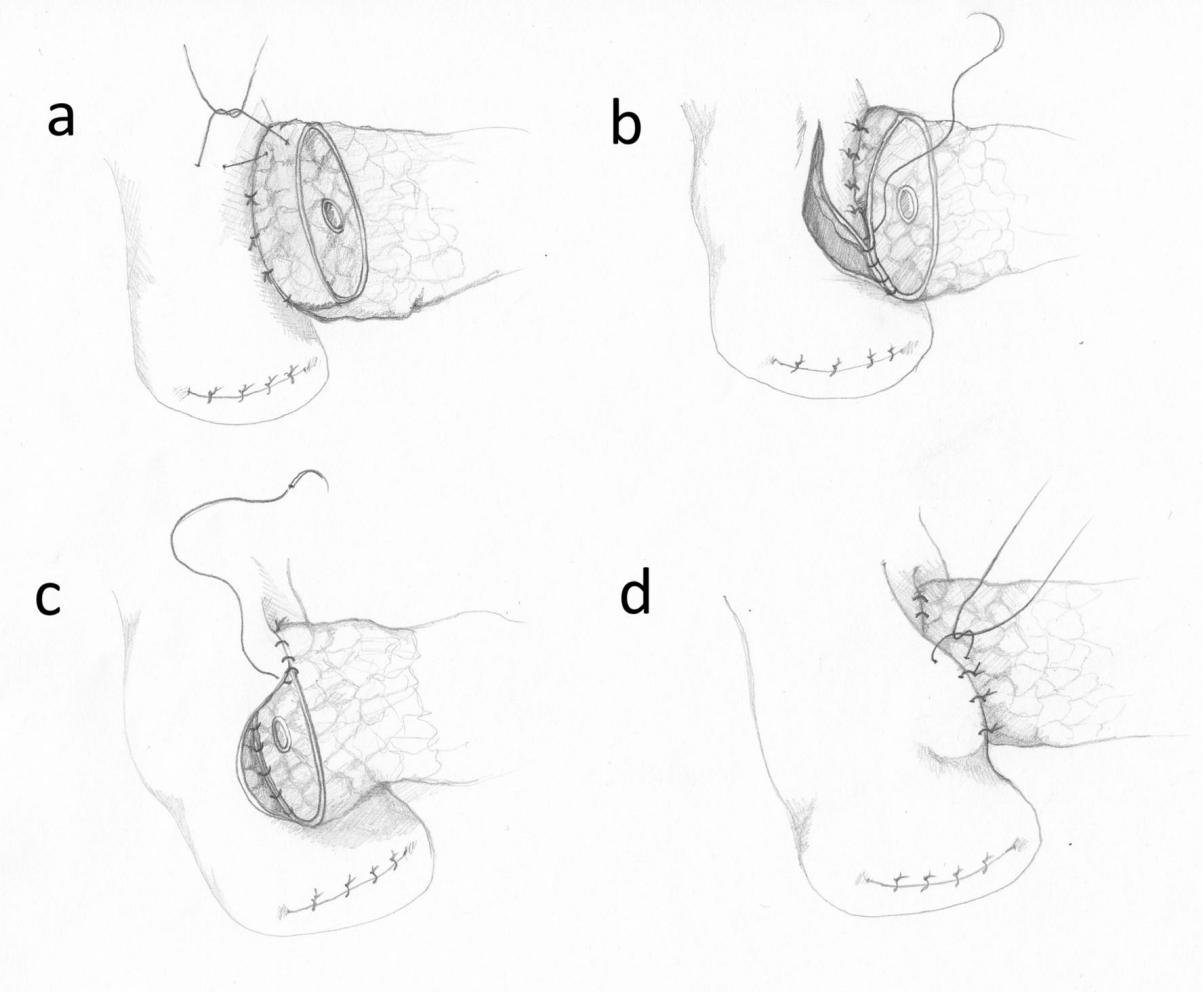
The conventional invagination technique. **a** The posterior external wall of the anastomosis. **b** The posterior internal wall. **c** The anterior internal wall. **d** The anterior external wall

The posterior external wall of the anastomosis is performed by placing 8 to12 interrupted 4–0 sutures between the posterior side of the pancreas and the jejunal wall. The sutures start 6–10 mm from the edge of the pancreatic stump, pass through the pancreatic capsule and parenchyma, a couple mm deep and about 10 mm long. Subsequently, the needle passes symmetrically through the seromuscular layer of the jejunum, perpendicularly to the long axis of the intestine. The sutures are tied only after they are all in place (Fig. 1a).

In the next step, the jejunal wall is cut longitudinally by electrocoagulation, along the sutures. The cut should be slightly shorter than the diameter of the pancreatic stump because of high elasticity of the intestine. The internal layer of stitches enters the pancreatic parenchyma in 1/3 of the stump’s diameter and passes to the capsule and through the full-thickness of the jejunal wall. The internal stitches should not cross the external suture layer (Fig. 1b). If the MPD is wide, the stitches may include its edge. If it is narrow, special attention must be paid to avoid ligation of the MPD. The internal layer of the sutures is continued around the pancreatic stump, from the posterior side towards the front (Fig. 1c). After the internal stitches (interrupted or continuous) are completed and tied, the external front sutures are performed. The needle passes as before, between the pancreatic capsule, 5–10 mm from the edge of the stump and through the seromucosal layer of the intestine. Tying of the external front suture layer causes invagination of the pancreatic stump into the jejunum and the external and internal suture layers move apart from each other (Fig. 1d). This last phase of the anastomosis is particularly difficult, because unskillful securing of the knots may tear the pancreatic capsule and cause a POPF.

### Modifications of the standard invagination technique

Recently, a “binding technique” proposed by Peng et al. has raised interest among surgeons [13]. In this method, the jejunal wall is pressed to the intussuscepted pancreatic stump by sutures placed similarly to a ligature. First, about 3 cm of the pancreatic stump must be isolated from the surrounding tissues. Corresponding 3 cm of the intestinal wall is everted by a few stitches and the mucosa of this part is cauterized or destroyed by 10% carbolic acid. Then, the pancreatic stump is attached to the jejunum by sutures placed on the edge of the everted mucosa. The sutures keeping the jejunum inside out are then removed, positioning the pancreatic stump inside the jejunum with the removed mucosa. The jejunum is carefully compressed by a ligature placed around the intestine, 1.5–2 cm from the proximal end of the intussuscepted pancreatic stump. The authors performed 227 anastomoses using this method (1996–2003) and did not observe any POPF. These astonishing results achieved by Peng et al. have not been replicated in any of the European or Asian centers using the same technique [14–16].

Many surgeons propose avoiding the second layer of sutures between the pancreatic capsule and the intestine. Kim et al. modified the Peng technique and after placing the stump 3 cm deep into the intestine they used only two transpancreatic U-sutures securing the upper and lower border of the jejunum, about 2 cm from the edge of the pancreatic stump. The sutures were tied using special square buttresses (TFE Polymer Pledget, Ethicon Inc.) [17]. Similarly, Li et al. used 3 overlapping transpancreatic U-sutures to secure the pancreatic stump dunked 3 cm deep into the intestine [18].

Kelemen et al. propose an end-to-side anastomosis performed by dunking the pancreatic stump 2–3 cm into the intestine with only 3 stitches. First, a purse-string suture is placed around the intestinal opening. In the next step, two U-sutures fix the end of the pancreatic stump deep inside the jejunum. Finally, the purse–string suture is tied so as to surround the intussuscepted pancreas by the intestinal seromuscular layer [19]. A nearly identical anastomosis was described by Li et al. with good results [20]. In another variation of Kelmen’s technique presented by Hashimoto et al., tying of the purse–string suture is preceded by 4 or 5 U-sutures fixing the pancreatic stump to the intestine with no stitches on the cut end of the pancreas [21].

The “serous touch” technique omits the external layer of stitches and 3 cm of the intestinal wall are intussuscepted into the lumen, doubling the intestinal wall and creating a cuff. Next, two U-sutures (starting from the outside of the intestine) are used to pull the pancreatic stump into the cuff, creating an end-to-end anastomosis. The adherence of 2–3 cm of the intestinal serosa to the pancreatic capsule should subsequently lead to prevention of pancreatic leakage [22].

Gupta et al. claimed that a single layer of 4–0 sutures between the pancreatic stump and the intestine is enough to prevent POPFs. In this anastomosis, the needle is inserted through the pancreatic capsule, then parenchyma and next through the whole intestinal wall. In the discussion, the authors emphasize that the single layer of the sutures minimizes the risk of pancreatic trauma and therefore the risk of POPF is reduced. However, the study was performed on a small group of 32 patients and a larger randomised study is required to confirm these results [23].

**Fig. 2:**
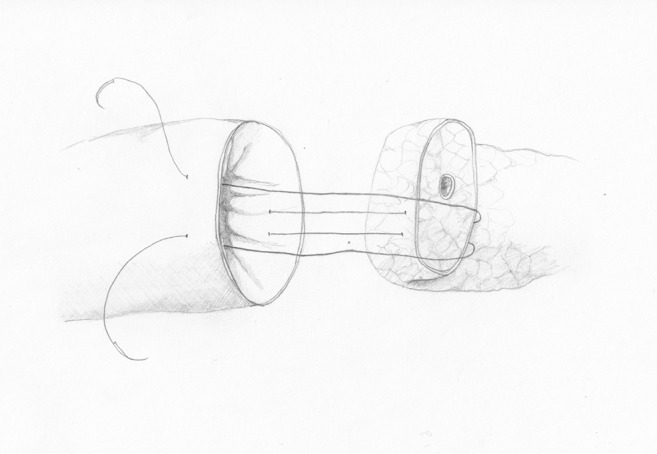
Chen’s U-suture

Chen XP et al. published a new technique of pancreaticojejunal end-to-end anastomosis with 2–4 single interrupted U-sutures (Chen’s U-suture), performed with double-armed sutures. The first needle passes from the outside to the inside of the posterior intestinal wall, about 1.5 cm from its cut edge. Then, it passes from the back to the front surface of the pancreas and again through the intestine, from the inside to the outside, about 1.5 cm from its cut edge. The second needle is led similarly about 1 cm from the first stitch. Finally, both ends of the suture are tied (Fig. 2) [24]. A similar technique was presented by Cho et al. for an end-to-side anastomosis with single interrupted mattress invaginating sutures, resulting in good outcomes in a group of patients with narrow MPD and soft pancreatic tissue [25]. An almost identical technique is also recommended by Korean authors [26] and another by Yang et al. in a middle segment pancreatectomy [27].

Yao et al. compared the incidence of the POPF in anastomoses performed with interrupted or continuous suture. Interrupted sutures were placed in 2–3 mm intervals in one layer. In the continuous suture group, the back wall of the anastomosis had a single layer of sutures and the anterior wall had two layers of continuous sutures. The outcomes were better in the continuous suture group [28]. Katoch et al. also used a technique of two 4–0 continuous sutures in an end-to-end anastomosis with good results [29]. Liu et al. performed the anastomosis in a single layer of continuous 5–0 double-armed sutures. In this technique, the needle enters the pancreatic stump 0.5 cm from its edge and it is passed through 50% of the pancreatic diameter, omitting the MPD which is not anastomosed. Then, the needle is led through the intestinal seromuscular layer. This technique is supposed to prevent creation of any free space between the intestine and the pancreatic stump [30].

Yang et al. presented another modification, called „the Colonial Wig” due to its appearance. First, the pancreas is invaginated by U-sutures into the antimesenteric side of the jejunum. Then, the closed end of the jejunum and its deferent loop are sutured by single interrupted stitches to the pancreatic trunk, thus securing the upper and lower corner of the anastomosis. The finishing touch is a sealing layer of interrupted sutures between the pancreatic capsule and the intestinal wall [10].

Morelli et al. presented the outcomes of 100 patients with a double-layer pancreaticojejunal anastomosis with small intestinal incision. The outer layer of this anastomosis is formed by interrupted mattress sutures placed about 10 mm from the cut end of the pancreas, between the pancreatic capsule and the seromuscular layer of the intestine. The internal layer is a continuous suture placed between the jejunal seromuscular layer and the edge of the pancreatic stump. After the back layer of the anastomosis is finished, a small incision is made in the intestine opposite to the MPD and the MPD is stented internally. No sutures are placed between the MPD and the intestinal incision. The authors of the study recommended this type of anastomosis for patients with high risk of POPF (soft pancreas, narrow MPD) [31].

## Duct-to-mucosa techniques

It seems that centers with a large-volume of pancreatic cases prefer to utilize an anastomosis where the MPD (and not only the whole pancreatic stump) is sutured directly to an opening in the jejunum—a so-called duct-to-mucosa technique [32, 33]. In this type of anastomosis, an external layer of sutures is performed by stitching either through the pancreatic capsule (in the Cattell-Warren technique) or transpancreatically (in the Blumgart and the Kakita techniques) and subsequently through the superficial layer of the jejunal wall.

### The Cattel-Warren technique

**Fig. 3:**
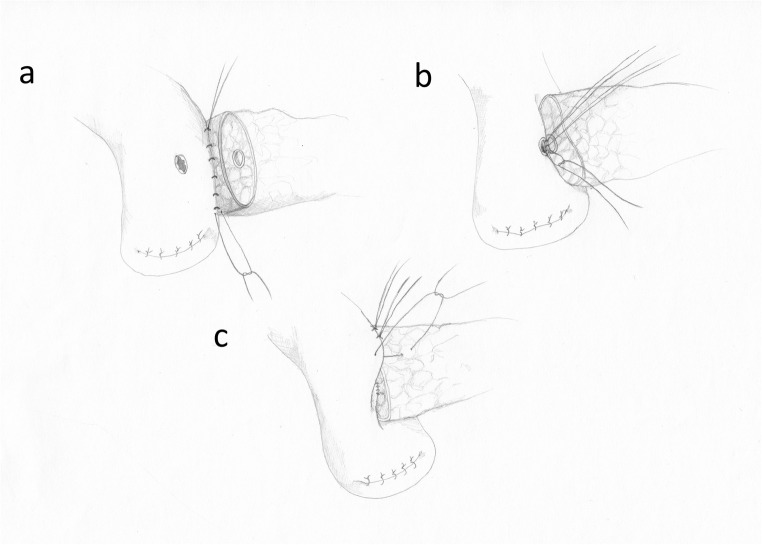
The duct to-mucosa anastomosis. **a** The posterior external wall. **b** The posterior interior wall. **c** The anterior external wall

The prototype of duct-to-mucosa anastomosis, still performed in many surgical centers, was proposed in 1956 by Cattell and Warren [34]. Initially, the tissues surrounding about 2 cm of the pancreatic stump are removed. Subsequently, single interrupted monofilament 4–0 sutures are placed through the posterior part of the pancreatic capsule (parallel to the axis of the pancreas, 1 cm from the cut edge) and through the seromuscular layer of the intestine. After 8–10 sutures are completed, they are carefully tied, forming the external layer of the posterior wall of the anastomosis (Fig. 3a). Then, the intestine is opened by electrocautery exactly in opposite position to the MPD. The cut should be of identical size as the MPD. Depending on the MPD diameter, 6–12 single interrupted monofilament 5–0 sutures are placed between the wall of the MPD (often including a part of the surrounding pancreatic tissue) and through the full wall of intestinal opening (Fig. 3b). Tying is performed from the back of the MPD towards the front. Finally, the external anterior layer of the anastomosis is performed with monofilament 4–0 single interrupted sutures between the pancreatic capsule and the seromuscular layer of the intestine (Fig. 3c) [34].

### Modifications of the duct-to-mucosa technique

**Fig. 4:**
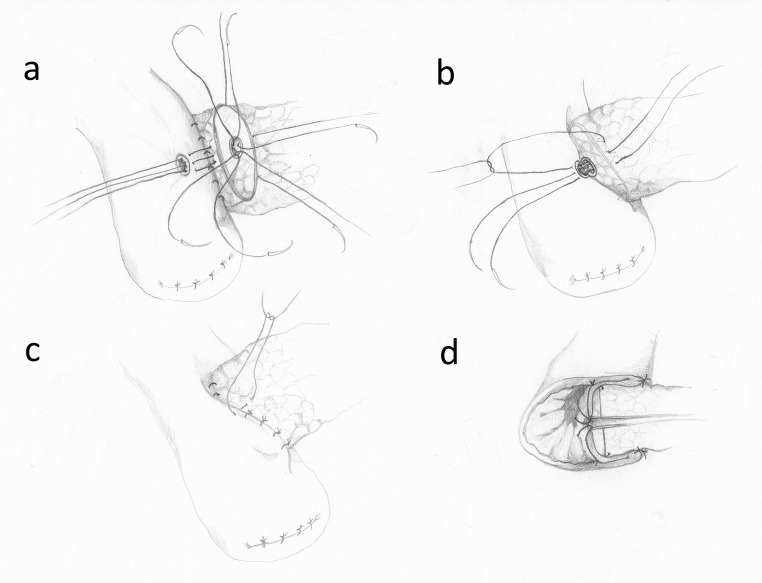
The modified Heidelberg technique. **a** The posterior external wall and four of the MPD stitches (8, 10, 12, and 2 o’clock). The 4 and 6 o’clock stitches were omitted for the clarity of the figure. **b** The posterior internal wall of the anastomosis tied. **c** The anterior external wall of the anastomosis tied. **d** The frontal section of the anastomosis

Torres et al. presented a modified version of the duct-to-mucosa anastomosis (the modified Heidelberg technique). First, three prolene 5–0 stitches are placed from the inside of the MPD, through its posterior wall and transpancreatically, exiting on the posterior surface of the pancreas. They are placed at 4, 6, and 8 o’clock position in the MPD. On the surface of the pancreas, the sutures should be 1 cm apart. Similar stitches are placed on the front wall of the MPD, at 10, 12, and 2 o’clock. All six sutures are suspended with a clamp and they are not tied at this point. Then, an external posterior running suture is performed with 4–0 prolene, starting from the edge of the pancreatic stump and through the seromuscular layer of the intestine. A 0.5-cm-wide opening is made in the intestine by electrocautery, opposite to the MPD (Fig. 4a). The three sutures previously placed at 4, 6, and 8 o’clock of the MPD are passed through the full thickness of the intestine, from outside to the inside and tied (Fig. 4b). An internal stent may be placed in the MPD at this point. The stitches from 10, 12, and 2 o’clock positions are now passed through the intestine, from the inside to the outside, and tied, forming the internal front layer of the anastomosis. Finally, a 4–0 running suture is placed between the pancreatic capsule and the seromuscular layer of the intestine (Fig. 4c, d). Additionally, the intestine is fixed with two hemostatic stitches, previously placed in the pancreas (before the removal of the pancreatic head) [35].

Papalampros et al. performed a similar anastomosis with 12 single interrupted 5–0 or 6–0 sutures for the MPD and performed the outer suture layer with one running double-armed stitch, with very promising results in a large group of patients [36].

Su et al. described a 3-layer duct-to-mucosa anastomosis. First, an oval-shaped opening the size of the pancreatic stump is cut in the seromuscular layer of the intestine, without cutting of the mucosa. The 4–0 PDS single interrupted stitches between the pancreatic capsule (0.5–1 cm from the cut edge) and the serosa of the intestine, close to the edge of the intestinal opening, are the first layer. For the second layer, similar stitches are used, placed between the pancreatic edge and the seromuscular layer of the intestine. For the third layer, a small incision is made in the intestinal mucosa and a duct-to-mucosa anastomosis is performed with 4 to 6 single interrupted 4–0 or 5–0 stitches [37].

Zhang et al. presented an anastomosis with a single layer of 6 interrupted monofilament double-armed 4–0 stitches. The first needle of the suture always goes from the inside of the MPD to the surface of the pancreatic stump, 0.5 cm from its cut edge. The second needle goes from the inside of the intestinal opening in the seromuscular layer of the intestine, on a distance similar to the radius of the cut pancreas. If the cut surface of the pancreas is presented as a 360° circle, then the stitches are 60° from each other. Additional U-sutures are placed on the upper and lower edge of the anastomosis, between the pancreatic capsule and the intestinal wall. Even though the examined group was too small (22 patients) to draw conclusions, the authors claim that their method reduces the POPF rate due to very good adhesion of the MPD and the pancreatic stump to the intestine. The number of stitches reduced to 6 provides less injury to the delicate pancreatic parenchyma [38]. Another publication showed that single layer sutures, going from the edge of the stump to the MPD prevent pancreatic fistula better than sutures passing only through a part of the pancreatic parenchyma [39]. The advantages of single- and double-layer anastomoses are about to be assessed in prospective randomized trials [40].

Kim et al. changed the order of placing sutures in a duct-to-mucosa anastomosis. Instead of starting with the external back layer, they suggest completing and tying the internal single interrupted sutures first (starting with the back wall of the anastomosis). This maneuver enables very good visualization of the MPD throughout the stitching process and there is more space for the needle. Only after the internal layer is ready, the second layer of sutures is performed, between the pancreatic capsule and the seromuscular layer of the intestine [41].

Some surgeons claim that a continuous stitch provides a more even distribution of force in the suture compared to single interrupted stitches and results in less tissue damage. This damage is crucial in “soft pancreas” anastomosis, where tying of the sutures may cause a leak of the pancreatic juice from microinjuries of the parenchyma.

A comparison between a 2-layer duct-to-mucosa anastomosis performed either with standard single interrupted sutures or with continuous sutures was performed by Ji et al. and also by Chen et al. The operative technique of the continuous suture includes 4–6 stitches (PDS 4–0) for the anterior and the posterior wall of the anastomosis. The needle is passed from the MPD, through the pancreatic tissue and through the full intestinal wall to the intestinal opening. The external layer is also performed with a continuous suture. In both studies, continuous sutures seemed to decrease the incidence of POPFs [42, 43].

### The Blumgart and the Kakita techniques

**Fig. 5:**
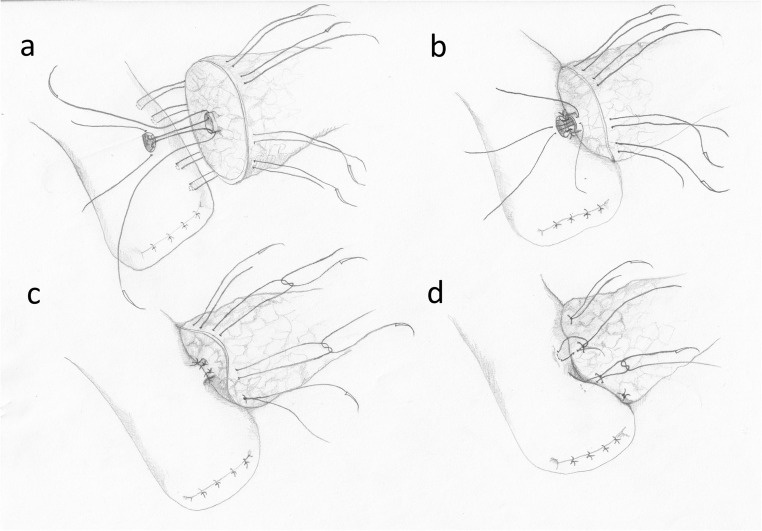
The Blumgart anastomosis. **a** The external layer of U-sutures. **b** The MPD anastomosis—the posterior sutures tied. **c** The finished MPD anastomosis and tying of the U-sutures. **d** The second tying of the U-sutures

Leslie Blumgart developed an anastomosis where the external layer consists of 4–8 U-sutures in intervals of ca. 0.75 cm. The needle is passed from the anterior to the posterior wall of the pancreas, a few mm from its cut edge. Then, through the seromuscular layer of the intestine, parallel to its axis, and again from the posterior to the anterior of the pancreas (Fig. 5a). These sutures are left untied and the duct-to-mucosa anastomosis is performed, usually with 6 single interrupted sutures (PDS 5–0). The number of sutures depends on the MPD size (Fig. 5b). In the next step, the U-sutures are carefully tied (Fig. 5c), the needles of these sutures are passed again through the seromuscular layer of the jejunum and tied for the second time (Fig. 5d), thus finishing the second layer of the anastomosis [44].

According to some studies, the Blumgart technique provides better early results than the Cattell-Warren technique (complication rate 17% vs 36%, POPF 4% vs 13%) [33]. These observations, however, require confirmation in recently launched randomized trials [45]. The Blumgart anastomosis is also effective in laparoscopic and robotic surgery [46–48].

**Fig. 6:**
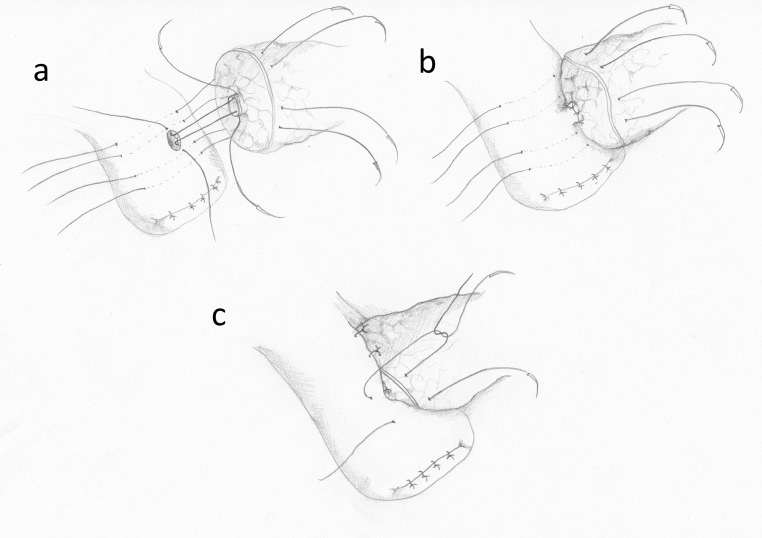
The Kakita technique. **a** The external layer of transpancreatic sutures. **b** The MPD anastomosis. **c** The external sutures—tying

Hirono et al. compared a modified Blumgart technique with the Kakita technique in a prospective randomized trial. In the modified Blumgart technique, only 1–3 U-sutures were used in order to reduce the possibility of pancreatic juice leakage and to enhance the perfusion of the pancreatic stump. The authors tied the U-sutures only once, after passing the needle through the seromuscular layer of the intestine. According to the authors, these modifications prevent pancreatic damage during tightening of the knots. The external layer in the Kakita method consists of 4 simple interrupted sutures placed transpancreatically and through the seromuscular layer of the jejunum (Fig. 6). There were no statistically significant differences between the two methods concerning the incidence of POPF [49]. Also, no significant difference between these two methods was found in a study by Kawakatsu et al., who examined the methods in a group of patients with “soft pancreas” [50].

Koijma et al. showed that the Blumgart technique combined with a so-called “complete packing method” of wound dressing reduces the occurrence of complications, including POPFs, after pancreaticoduodenectomy. The study included 374 patients and 3 types of anastomoses: the Cattell-Warren technique, a modified Kakita method and a modified Blumgart technique. In the Blumgart technique, the abdomen was thoroughly washed before closing (10 L of saline), an internal stent was inserted into MPD and tight dressing was applied to the abdominal wound and around the drains. The dressing was removed only after evacuation of the drains. POPF type B and C was noted in 20 out of 103 patients in the Cattell-Warren technique group, 49 out of 170 in the Kakita technique group and only 3 out of 101 in the Blumgart technique group [51].

Wang et al. modified the Blumgart technique for laparoscopic surgery of the pancreas with a non-dilated MPD. After tying two of the transpancreatic U-sutures as an external layer, identical to the Blumgart technique, two other stitches are placed transpancreatically as the second, internal layer. They exit on the posterior wall of the pancreatic stump and pass through the full intestinal wall, parallel to its axis and below the intestinal cut. Tying of these sutures presses the intestinal wall to the pancreatic stump below the MPD. On the front wall of the anastomosis, the transpancreatic U-sutures exit on the cut wall of the pancreatic stump. When these sutures are tied, the front wall of the intestine is pressed to the front wall of the pancreatic stump. The intestinal cut and the MPD are not anastomosed, but an internal stent is applied in all cases. The anastomosis is finished by an external row of sutures placed between the front surface of the pancreas and the seromuscular layer of the intestine [52].

## Conclusions

Despite the large number of publications on pancreatic fistula after pancreaticoduodenectomy in recent years, it still remains a major challenge for surgeons [10]. No surgical technique gives any advantage in eliminating the risk of pancreatic fistula [11]. However, a duct-to-mucosa anastomosis where mucosa is performed with single, synthetic absorbable stitches (PDS 4.0–5.0) is mostly advised [32, 33]. It should be assumed that the pancreatic-intestinal anastomosis must be fitted to the personal preferences and experience of the surgeon and any new method leading to lower percentage of POPFs is welcome in pancreatic surgery.
